# Automatic ^1^H-NMR Screening of Fatty Acid Composition in Edible Oils

**DOI:** 10.3390/nu8020093

**Published:** 2016-02-16

**Authors:** David Castejón, Pascal Fricke, María Isabel Cambero, Antonio Herrera

**Affiliations:** 1 Centro de Asistencia a la Investigación de Resonancia Magnética Nuclear y de Resonancia de Spin Electrónico (CAI de RMN y RSE), Universidad Complutense de Madrid, 28040 Madrid, Spain; dcastejon@pluri.ucm.es; 2 Department of Molecular Biophysics, Leibniz-Institut für Molekulare Pharmakologie, 13125 Berlin, Germany; fricke@fmp-berlin.de; 3 Department of Nutrition, Bromatology and Food Technology, Veterinary Faculty, Universidad Complutense de Madrid, 28040 Madrid, Spain; icambero@ucm.es; 4 Department of Organic Chemistry, Chemistry Faculty, Universidad Complutense de Madrid, 28040 Madrid, Spain

**Keywords:** NMR spectroscopy, edible oil, fatty acid composition, iodine value, automatic analysis, MestreNova, TopSpin

## Abstract

In this work, we introduce an NMR-based screening method for the fatty acid composition analysis of edible oils. We describe the evaluation and optimization needed for the automated analysis of vegetable oils by low-field NMR to obtain the fatty acid composition (FAC). To achieve this, two scripts, which automatically analyze and interpret the spectral data, were developed. The objective of this work was to drive forward the automated analysis of the FAC by NMR. Due to the fact that this protocol can be carried out at low field and that the complete process from sample preparation to printing the report only takes about 3 min, this approach is promising to become a fundamental technique for high-throughput screening. To demonstrate the applicability of this method, the fatty acid composition of extra virgin olive oils from various Spanish olive varieties (arbequina, cornicabra, hojiblanca, manzanilla, and picual) was determined by ^1^H-NMR spectroscopy according to this protocol.

## 1. Introduction

Over the past years, Nuclear Magnetic Resonance (NMR) has proven to be an efficient technique to determine the composition of fatty acids in a simple and quick manner [1,2]. There is a high demand for this information as the fatty acid profile is used to classify the quality of edible oils [3] and as an indicator for the detection of adulteration [4]. In the last years different authors applied the ^1^H-NMR spectroscopy for the study of extra virgin olive oils (EVOO) focusing on differentiate EVOO according to the aldehydes present and the combination of ^1^H-NMR with multivariate statistical approach [5,6,7]. Furthermore, printing the composition on the label of edible oils is obligatory in the European Union since December 2014 (*Regulation 1169/2011*) [8]. 

Traditionally, NMR has been an analytical technique used for the verification and elucidation of molecular structures and for the determination of purity. But due to the needs of emerging fields like metabolomics, NMR has evolved rapidly to be a screening technique for the analysis of mixtures. This development has been facilitated by the technical advances in the field of automatic sample changers, the quality of NMR spectrometers, and by the development of new software for spectral data processing. The main applications for NMR are drug discovery [9] and clinical diagnostics [10]. Developing a totally automated system, which comprises the preparation, the acquisition of spectral data, the processing, the analysis, and the interpretation of the data, these advances in mixture analysis have recently been successfully transferred to the analysis of fruit juice [11,12].

In this work, we describe the evaluation and optimization needed for the automated analysis of vegetable oils by low-field NMR to obtain the fatty acid composition (FAC). To achieve this, two scripts, which automatically analyze and interpret the spectral data, were developed. To ensure a widespread applicability, they were written for the two most important software programs used for the analysis of NMR data (Bruker TopSpin, Rheinstetten, Germany and Mestrelab MestReNova, Santiago de Compostela, Spain). The objective of this work was to drive forward the automated analysis of the FAC by NMR. Due to the fact that this protocol can be carried out at low field (300 MHz) and that the complete process from sample preparation to printing the report only takes 3–4 min, this approach is promising to become a fundamental technique for high-throughput screening in both, the industry and in food control laboratories.

## 2. Results and Discussion

### 2.1. Optimization and Evaluation of the FAC by NMR Experiments at 300 MHz

This work is based on previous results [1] which proved the accuracy of the determination of the fatty acid composition using ^1^H-NMR spectra acquired at 500 MHz (in less than a minute) in comparison with the standard method (determination by gas chromatography with flame ionization detection, GC-FID [13]). In this work, we evaluate the possibility to achieve similar results at an even lower magnetic field (7 T; 300 MHz). These instruments are more widely available as they have less demanding requirements in terms of hardware installation and are considerably cheaper. A better availability of the instruments to carry out the protocol presented here would also result in a more widespread applicability of this approach as well in the field of food control laboratories as in food industry. To carry out this evaluation, the same acceptance criteria for the NMR analyses as in previous works was used (smallest peak to be integrated should present a signal to noise ratio of at least 250:1, spectral resolution of at least the half of the line-width at half-height). According to these criteria, the parameters described in the “Materials and Methods” section were selected (NS = 8 resulted in a S/N = 567.33 ± 17.31 for the signal B in the picual oil, whose signal was selected as it is characteristic for linolenic acid and only present in certain oils; the line-width at half-height of the TMS signal was 0.68 Hz, the spectral resolution with the selected parameters was 0.11 Hz).

**Figure 1:**
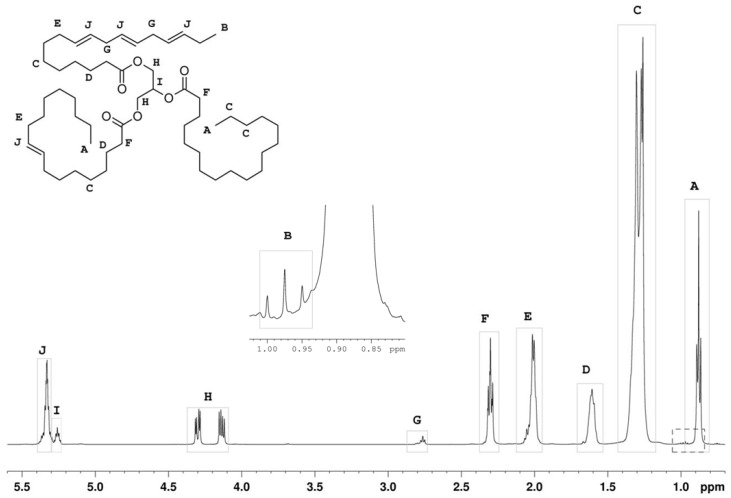
^1^H-NMR spectrum of olive oil with corresponding assignment of the signals of the glycerol unit and the fatty acid chains. The region from 1.03 to 0.82 ppm is shown in an expansion. The figure also shows the structure and nomenclature used for the glycerol moiety and the different fatty acids protons.

**Figure 2:**
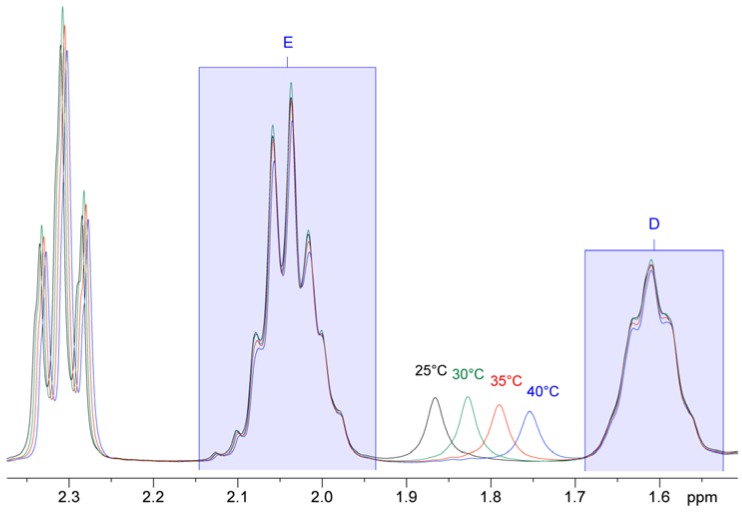
Temperature series of 300 MHz ^1^H-NMR spectra of soy oil in CDCl_3_ referenced to internal TMS. The spectra were acquired at 4 different temperatures and the results were overlaid to illustrate the dependence of the water signal chemical shift on the sample temperature.

As a consequence of changing to a lower magnetic field, certain factors needed to be taken into account when selecting the integration regions: The influence of the ^13^C satellites changes, as, for example, working at 300 MHz, the ^13^C satellite of signal A does not influence the integral of signal B as it is outside its region, which is not the case at higher fields [1,14]. Furthermore, the effect of the signal of the residual water was evaluated. As this signal is temperature dependent, the experiments were carried out at different temperatures to monitor its change in chemical shift. Figure 2 shows the variation of the residual water signal at different temperatures. To avoid any influences on the integrals, a temperature of 30 °C was chosen. Figure 1 shows the ^1^H-NMR spectrum acquired using the aforementioned conditions and the assignment of the fatty acid signals. 

The fatty acid composition was carried out according to the formulae described in M&M and it was observed that the results were satisfying (no significant differences—*p* < 0.05—in comparison with the results obtained from the same oils at 500 MHz using the previously described method). Then it was evaluated if the automatic analysis permits to obtain the same values for the FAC as those obtained from manual analysis (where mostly shimming is the determining factor). The obtained results did not show significant differences (*p* < 0.05) between the manual and automatic analysis using the same samples. The differences between the results from the manually processed spectra (phase correction, baseline correction, referencing to 0 ppm) and the automatically processed spectra were not significant (*p* < 0.05), either.

Once it was proven that the results of the calculation of the FAC were satisfying at low field and using automatic acquisition and processing, programs were developed to carry out the automation. The automatic process starts after placing the samples into the autosampler and ends with displaying the final report containing the FAC for each of the analyzed oils. 

### 2.2. Automated Analysis of the FAC via Automatic ^1^H-NMR Acquisition and Analysis at 300 MHz

The aim was that a possibly large amount of acquired spectra (for example via an autosampler) should be processed and analyzed (integration, calculation of fatty acid composition) in a completely automatic manner. The desired result is a table showing the fatty acid composition (in %) for each measured sample. By achieving this, a high throughput of samples can be obtained and the whole process would require minimal attendance.

It was chosen to write scripts that carry out the whole automation process. The scripts were written for the two most widely used programs for NMR data processing: MestreNova [15] and TopSpin [16]. 

#### 2.2.1. MestReNova Script

The NMR processing program MestReNova supports extending its capabilities and implementing automated workflows via an ECMAScript-based Application Programming Interface (API) to access the core functionality of the program and to generate user output.

**Figure 3:**
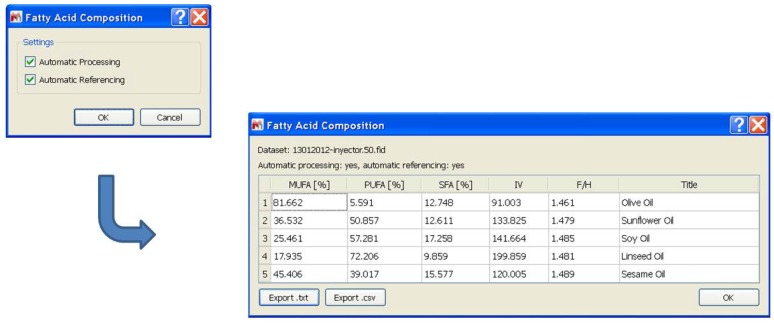
Result window shown in MestReNova after completing the automatic analysis.

This interface was used to write a script, which provides the automatic analysis function for proton spectra of edible oils using MestReNova as a basis. This extends the availability of the automated fatty acid analysis to all platforms MestReNova runs on (Windows, GNU/Linux, Mac OS X) and to all data formats MestReNova can read (all major vendors/models/formats). This versatility also makes this software one of the most widely used worldwide [15,17]. The script expects all to-be-processed spectra to be opened; it does not matter whether they are on different pages or within the same page, they only must be part of the same document. The first dialog upon executing the script expects the user to specify whether the spectra shall be automatically processed (*i.e.*, automatic phase correction and automatic baseline correction) and whether the spectra shall be automatically referenced (against TMS). Both options are by default activated and can be unchecked, for example if manual corrections are desired or if the spectra were acquired without TMS added. After accepting this dialog, all the processing, the integration, and the calculation are done automatically and the results are shown in a dialog in form of a table (Figure 3). 

The results table generated by this script also contains the iodine value (IV) of all oils by evaluating the signal J (see M&M). The IV shows the degree of unsaturation of the oils and often has been used to predict the chemical and physical properties of fats and oils, such as oxidative stability and melting points [18]. Additionally, the table contains the F/H ratio. This ratio has been proposed as a single way to validate the approximation used to calculate the FAC [1]. Its value should be close to 1.50, since the signal F (CH_2_ in alpha-position to the carboxylic acid) represents six protons and the signal H (CH_2_ from *sn-1,3* TG) represents four protons. A value different from 1.50 can be explained by a higher concentration of diglycerides, monoglycerides, and/or free fatty acids. That is why the F/H ratio indicates whether the approximations are correct and therefore produce trustworthy FAC values. Anomalous values for F/H would indicate that this is not the case and that the FAC values are possibly erroneous.

The scripts (MestReNova and TopSpin) were evaluated with a set of edible oils (olive, sunflower, soy, linseed, and sesame oils) that have a largely differing fatty acid composition. The results have shown no significant differences to those obtained after manual FAC calculation. The scripts also offer the user the possibility to export the calculated data as a human-readable text file (.txt) or as comma-separated-values (.csv). The MestReNova script is available in the on line supplementary data (Section S1).

#### 2.2.2. TopSpin AU Program

In our Bruker-equipped NMR laboratories the spectra acquisition software used is TopSpin. This software allows the implementation of custom automatization workflows via several different programming interfaces like the Python interface, the AU (automatic) programming interface (AU) or traditional macros. AU programs are compatible with a wide range of TopSpin versions (from the earliest to the newest) and allow a high level of customization and interaction with the user. Because of these reasons and because of the widespread availability of TopSpin, this programming interface was also chosen as a basis for the implementation of the automatic analysis function for proton spectra of edible oils. The Topspin AU program is described in supplementary data (Sections S2 and S3).

#### 2.2.3. Total Automated Analysis of the FAC

**Figure 4:**
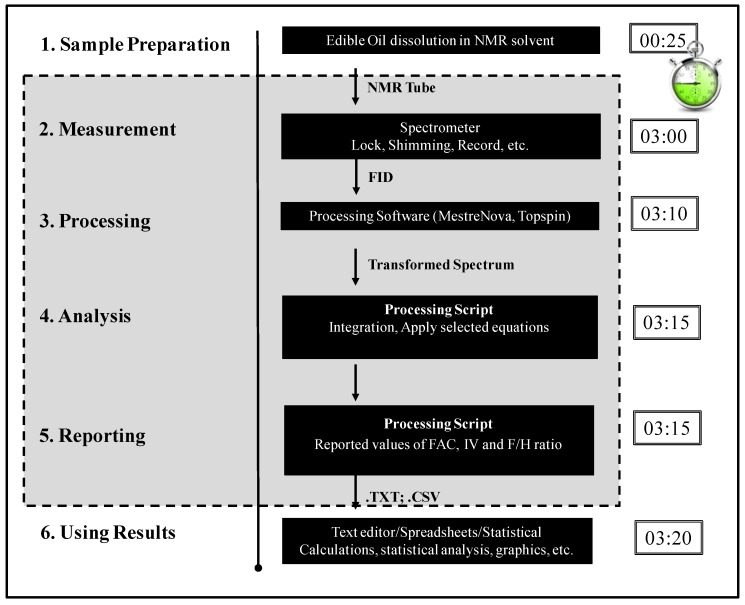
Scheme illustrating the general workflow for the automatic edible oil analysis: (**1**) Performing the (quantitative) NMR experiment itself and recording the FID; (**2**) Transforming the FID with FT to a spectrum and applying all associated processing steps (apodization, phasing, baseline correction, *etc.*); (**3**) Using the processing script to extract the information from the spectra; (**4**,**5**) Using the information provided by the processing script for plotting or further analysis, such as statistics; (**6**) Export the reported values of FAC, IV and F/H ratio to other programs.

Figure 4 shows a scheme illustrating the whole process from sample preparation to exporting the obtained data and includes estimations on the duration of each of the individual steps. With the protocol proposed here, it is possible to obtain the FAC, the IV, and the F/H ratio in about 3 min. The only not yet automated steps are the sample preparation and the post-processing of the data (statistics, diagrams). Although these steps can be automated, this was out of the scope of the work presented here. The grey box in Figure 4 represents the automated workflow.

### 2.3. Automatic Characterization of the FAC in Extra Virgen Olive Oils (EVOO)

The fatty acid composition of extra virgin olive oils from various Spanish olive varieties (arbequina, cornicabra, hojiblanca, manzanilla, and picual) was evaluated by the ^1^H-NMR automatic analysis described above. The data indicated that all the investigated olive oils had the expected EVOO fatty acid composition, with oleic acid being the most abundant for all the varieties (Table 1). According to the literature [19], the EVOO from arbequina has the highest PUFA concentration while the EVOO from picual has the lowest. The results did not show significant differences between the intra-sample standard deviation (3 consecutive experiments using the same sample) and the inter-sample standard deviation (3 samples of each oil acquired randomly).

## 3. Materials and Methods 

Several types of oils were used during the study. Firstly, a set of commercial oils with differing FAC was selected: olive oil, sunflower oil, linseed oil, sesame oil, and soy oil. Secondly, a set of extra virgin olive oils from differing Spanish olive varieties was chosen: arbequina, cornicabra, hojiblanca, manzanilla, and picual. These samples were obtained from different olive oil companies (Hojiblanca, Casas de Hualdo, and Finca la Redonda). 

### 3.1. NMR Analysis

For the sample preparation of all types of edible oils, only the addition of deuterated solvent was required as a single step. 600 μL of CDCl_3_ (containing 1% TMS, Tetramethylsilane) and 50 μL of oil were placed in a 5 mm NMR tube and mixed thoroughly during 10 s. The spectra were acquired using a Bruker DPX 300 MHz NMR spectrometer, equipped with a BACS-60 robotic autosampler (which allows for fully automated analysis of up to 60 samples at a time) and a 5 mm Z-gradient QNP probe. For the acquisition, 32 K complex points were recorded, the spectral width was set to 12 ppm, the frequency offset was set to 5 ppm, the recycle delay was set to 4 s, the acquisition time was 4.56 s, the excitation pulse was a 90° hard pulse, the number of scans was set to 8 and the number of dummy scans was equal to 2. The total experimental time was 1 min and 26 s. A temperature of 30 °C was chosen for the experiments. The data were acquired automatically using the software ICON-NMR (Bruker BioSpin, Rheinstetten, Germany).

The resulting spectra were processed manually and automatically with the software MestreNova 9.0.1 (Mestrelab Research SL, Santiago de Compostela, Spain) and Topspin 2.1 (Bruker Biospin, Rheinstetten, Germany). No window functions were applied. The chemical shift scale was referenced using the signal of the TMS (0 ppm).

### 3.2. Fatty Acid Composition

The percentage of fatty acids was determined using the integrals of certain resonances in the ^1^H-NMR spectrum. The following equations developed in previous work [1] were used:

[SFA = 4 × A + 4 × B − 3 × E/6 × F](1)

[MUFA = 4 × B + 3 × E − 6 × H/6 × F](2)

[PUFA = 3 × G − 2 × B/3 × F](3)

In these equations, SFA refers to Saturated Fatty Acids, MUFA to Mono-Unsaturated FA, PUFA to Poly-Unsaturated FA, and the letters A-J to the NMR oil signals described in Figure 1. To calculate the total FA value, these equations consider that each acyl chain contains two methylenic protons in α-position to the carboxyl group (signal F). 

### 3.3. Determination of the Iodine Value

The determination of the iodine value (IV) was based on the ^1^H-NMR data by a previously developed approach [20] which proved that the iodine value is related to the percentage of olefinic protons (%OP) according to the equation:

[IV = 10.54 + 13.39 × %OP](4)

The percentage of olefinic protons (%OP) in the oil can be directly determined from the area of signal J in Figure 1.

## 4. Conclusions

It could be shown that NMR is a powerful tool to characterize edible oils in many different ways:

^1^H-NMR spectra allow the reliable quantitation of the fatty acid content with respect to monounsaturated fatty acids (MUFA), polyunsaturated fatty acids (PUFA), and saturated fatty acids (SFA). This process is so straightforward that even a completely automatic processing and analysis of the spectra is possible. A MestReNova script and a TopSpin AU program were created to automatically derive these values for a (possibly) large set of raw NMR data. Both scripts ensure compatibility on many different platforms. 

It can be concluded that NMR spectroscopy surpasses the established investigation methods (gas chromatography) for vegetable oils with respect to analysis time and sample preparation. Its use can ease and improve the characterization process significantly.

## Figures and Tables

**Table 1:** Mean values and standard deviations of the fatty acid compositions (%) of the different Spanish extra virgin olive oils (EVOO).

-		MUFA (%)	PUFA (%)	SFA (%)	IV	F/H
**Arbequina**	Sample 1	77.58 ± 0.05	8.19 ± 0.02	14.23 ± 0.03	91.91 ± 0.03	1.48 ± 0.00
	Sample 2	77.52 ± 0.10	8.25 ± 0.07	14.23 ± 0.03	92.03 ± 0.02	1.48 ± 0.00
	Sample 3	77.63 ± 0.05	8.26 ± 0.03	14.11 ± 0.04	92.15 ± 0.08	1.47 ± 0.00
	Mean value	77.58 ± 0.09	8.23 ± 0.04	14.19 ± 0.06	92.03 ± 0.10	1.47 ± 0.00
**Cornicabra**	Sample 1	85.16 ± 0.21	4.05 ± 0.15	10.78 ± 0.06	89.70 ± 0.06	1.47 ± 0.00
	Sample 2	85.03 ± 0.13	4.12 ± 0.10	10.86 ± 0.04	89.80 ± 0.11	1.47 ± 0.00
	Sample 3	85.53 ± 0.20	3.84 ± 0.15	10.63 ± 0.05	89.63 ± 0.04	1.47 ± 0.00
	Mean value	85.24 ± 0.27	4.00 ± 0.18	10.76 ± 0.09	89.71 ± 0.10	1.47 ± 0.00
**Hojiblanca**	Sample 1	84.30 ± 0.08	6.76 ± 0.03	8.94 ± 0.05	93.35 ± 0.02	1.48 ± 0.00
	Sample 2	84.06 ± 0.08	6.92 ± 0.10	9.02 ± 0.05	93.20 ± 0.01	1.48 ± 0.00
	Sample 3	84.25 ± 0.10	6.90 ± 0.07	8.85 ± 0.04	93.16 ± 0.03	1.47 ± 0.00
	Mean value	84.20 ± 0.10	6.86 ± 0.08	8.94 ± 0.05	93.24 ± 0.08	1.48 ± 0.00
**Manzanilla**	Sample 1	82.76 ± 0.08	3.13 ± 0.03	14.11 ± 0.06	85.88 ± 0.08	1.47 ± 0.00
	Sample 2	82.28 ± 0.13	3.39 ± 0.12	14.33 ± 0.06	86.16 ± 0.10	1.47 ± 0.00
	Sample 3	82.55 ± 0.09	3.22 ± 0.08	14.23 ± 0.01	86.05 ± 0.02	1.47 ± 0.00
	Mean value	82.53 ± 0.11	3.25 ± 0.09	14.22 ± 0.06	86.03 ± 0.12	1.47 ± 0.00
**Picual**	Sample 1	85.13 ± 0.13	2.45 ± 0.10	12.42 ± 0.04	89.42 ± 0.05	1.48 ± 0.00
	Sample 2	85.07 ± 0.04	2.50 ± 0.05	12.43 ± 0.01	89.37 ± 0.04	1.48 ± 0.00
	Sample 3	85.04 ± 0.06	2.52 ± 0.05	12.43 ± 0.00	89.51 ± 0.04	1.48 ± 0.00
	Mean value	85.08 ± 0.08	2.49 ± 0.07	12.43 ± 0.02	89.43 ± 0.07	1.48 ± 0.00

MUFA: monounsaturated fatty acids; PUFA: polyunsaturated fatty acids; SFA: saturated fatty acids; IV: iodine value; F/H: signal F/signal H ratio.

## Supplementary Materials

The following are available online at http://www.mdpi.com/2072-6643/8/2/93/s1, Section S1: The MestReNova script, Section S2: Topspin AU program, Section S3: Topspin AU program description.

## Abbreviations

The following abbreviations are used in this manuscript:

EVOO: Extra Virgin Olive Oils
FAC: Fatty Acid Composition
F/H: Signal F/Signal H ratio
IV: Iodine Value
MUFA: Monounsaturated Fatty Acids
NMR: Nuclear Magnetic Resonance
OP: Olefinic Protons
PUFA: Polyunsaturated Fatty Acids
SFA: Saturated Fatty Acids
TMS: Tetramethylsilane
