# Supplementary material for: Automatic 1H-NMR Screening of Fatty Acid Composition in Edible Oils
Source: Nutrients. 2016 Feb 16;8(2):93. doi: 10.3390/nu8020093 (PMC4772056; doi:10.3390/nu8020093)
Supplement: Supplementary file 1 [file nutrients-08-00093-s001.docx]

Supplementary Materials: Automatic ^1^H-NMR Screening of Fatty Acid Composition in Edible Oils

David Castejón, Pascal Fricke, M. Isabel Cambero and Antonio Herrera

Section S1: MestReNova script

The MestReNova script is available in [1].

Section S2: Topspin AU program

The Topspin AU program is available in [1].

Section S3: Topspin AU program Description

TopSpin AU programs are programs written using the C-language and can contain usual TopSpin commands in the form of macros. They allow access to the acquired data and can be used to process and change them; furthermore it is possible to interact with the user to influence the behavior of the program or to pass commands/parameters to the program. AU programs can be executed by simply entering the name of the program in the TopSpin command line and are compiled to form executable binaries whenever they are executed for the first time or their underlying source code gets changed. The latter, however, is a transparent process and does not require any special user interaction.

The program is initiated using the command *multi_calcfa*. It expects the spectra (*i.e.*, oil samples) to be stored in subsequent experiment numbers (EXPNO) of a dataset with the same name. This is the standard situation if spectra were acquired using an autosampler. The program asks the user to enter the first experiment number which is supposed to be processed (Figure S1A). Furthermore, it asks for the total of number of experiments which shall be processed (Figure S1B) and the increment between subsequent experiment numbers (Figure S1C). The latter takes into account that, using an autosampler, the experiment numbers between two subsequent samples are increased to the next tens, so that the increment is in many cases 10.

| 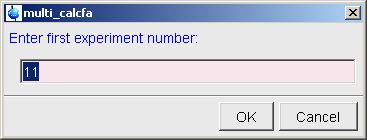 | 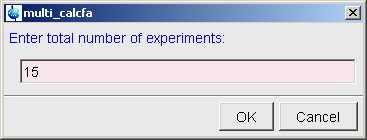 |
| --- | --- |
| (**A**) | (**B**) |
| 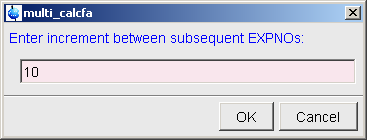 | |
| (**C**) | |

**Figure S1.** Dialogs of the TopSpin AU program needed for the user input. (**A**) First EXPNO to be processed; (**B**) Total number of EXPNOs to be processed; (**C**) Increment between EXPNOs.

The following steps of the execution of the program work without any user attendance and are completed within seconds. In the pdata/1/folder of the first experiment number two files are written: *multi_calcfa_result.txt* and *multi_calcfa_result.csv*. The first is a human readable text file which is also presented after the successful completion of the AU program (see Figure S2). It contains a table with all *processed* experiment numbers, the fatty acid concentrations and the corresponding title of the sample. The latter is a comma separated value file which contains the same results, but it can be easily imported by programs like Origin *or* Excel to ease further work with the obtained data and to assure a greater compatibility.


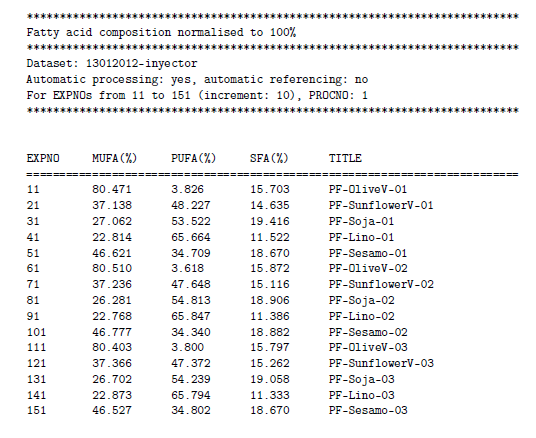


**Figure S2.** Sample output taken from the generated .txt-file of the TopSpin AU program. Here, 15 samples of different oils (olive, sunflower, soy, linseed and sesame oil) were analysed.

Besides this “standard workflow”, the program supports three command line arguments, which can simply be appended to the name of the program to take effect. The first one is “*noproc*”, which deactivates all automatic processing. In this case, the program expects all datasets to be already fully processed. This is especially handy if one wants to achieve better results than using the automatic phase and baseline correction algorithms. The second one is “*nosref*”, which suppresses the automatic chemical shift referencing to the TMS peak. This is useful if the computer used to process the spectra is not controlling the spectrometer and does therefore not have a valid lock-table available, or if the spectra were acquired without TMS added. The last command-line argument is simply represented by an integer number. This number refers to the processing number which is supposed to be used.
If none is given, 1 is the default. The purpose of this argument is to give the user the possibility to store the automatic processing of the AU program in a higher processing number (PROCNO) to avoid that manually processed data is overwritten; or to manually process data using a higher processing number so that different results can be counter-checked.

The AU program automatically checks the user input and the data to avoid misbehavior and to inform the user on what input is wrong and what kind of input is expected instead. The calculated percentages are normalized to sum up to 100%.

References

1. Google Drive. Available online: https://drive.google.com/drive/folders/0B0Zu2ZtVnB_hdFM5LUg4eGQz MGM (accessed on 4 February 2016).
